# Association of obstructive sleep apnea with risk of lung cancer: a nationwide cohort study in Korea

**DOI:** 10.1038/s41598-024-63238-x

**Published:** 2024-05-29

**Authors:** Jaeyoung Cho, Soomin Jo

**Affiliations:** 1 Division of Pulmonary and Critical Care Medicine, Department of Internal Medicine, Seoul National University Hospital, 101 Daehak-ro, Jongno-gu, Seoul, 03080 Republic of Korea; 2 Department of Internal Medicine, Seoul National University College of Medicine, Seoul, Republic of Korea; 3 Division of Pulmonary and Critical Care Medicine, Department of Internal Medicine, Dongguk University Ilsan Hospital, Goyang, Republic of Korea

**Keywords:** Obstructive sleep apnea, Lung cancer, Risk factors, Population-based, Risk factors, Sleep disorders, Lung cancer

## Abstract

Current knowledge regarding the relationship between obstructive sleep apnea (OSA) and the risk of lung cancer is limited. This study aimed to evaluate associations between OSA and the incidence of lung cancer based on the Korean National Health Information Database. The study outcome was the incidence of newly diagnosed lung cancer, and a Cox proportional hazards model was used for analysis. A total of 181,070 adult patients newly diagnosed with OSA between 2011 and 2018 were matched with those without OSA by up to 1:5 propensity score matching based on age and sex. During follow-up over (mean ± standard deviation) 9.1 ± 2.0 years, 2614 incident cases of lung cancer were identified. The incidence rate was 39.51 per 100,000 person-years in the OSA group, and 24.93 per 100,000 person-years in the control group. After adjusting for income and the presence of comorbidities, the association remained significant (hazard ratio [HR] 1.95, 95% confidence interval [CI] 1.74–2.18, *p*-value < 0.001). The adjusted HR for incident lung cancer was 2.14 (95% CI 1.69–2.70) in female patients with OSA, and 1.90 (95% CI 1.67–2.16) in male patients with OSA. The risk of incident lung cancer increased with age, with a HR of 2.99 (95% CI 2.46–3.64) in those aged ≥ 65 years. This nationwide study showed an independent association between OSA and an increased risk of lung cancer in the Korean population.

## Introduction

Obstructive sleep apnea (OSA) is a prevalent condition characterized by repeated episodes of partial or complete obstruction of the upper airway during sleep, leading to disrupted sleep and intermittent hypoxia^1,2^. It is increasingly recognized as a public health concern owing to its association with various comorbidities, including cardiovascular diseases, metabolic syndrome, and neurological disorders^1,2^.

While substantial research has revealed a link between OSA and several health issues, the relationship between OSA and cancer, particularly lung cancer, remains debatable^3^. Lung cancer, a leading cause of cancer-related mortality worldwide, has multifactorial etiology involving genetic, environmental, and lifestyle factors^4^. Hypoxia, a common feature in the tumor microenvironment, is known to influence tumor growth, angiogenesis, and resistance to therapy^5^. Thus, the potential role of OSA, with its hallmark intermittent hypoxic episodes, has been suggested in the context of lung carcinogenesis.

Current literature regarding the associations between OSA and the risk of lung cancer is often limited by a low incidence of lung cancer. Most studies have used the incidence of all cancers as the outcome measure and showed subgroup analyses evaluating the associations with specific types of cancer^6–9^. However, subgroup analyses may suffer from a lack of statistical power if there is an insufficient number of events^10^. In addition, most studies on OSA and lung cancer have been conducted in Western countries. Therefore, we aimed to evaluate associations between OSA and the incidence of lung cancer based on the Korean National Health Information Database (NHID).

## Methods

### Study design and population

We utilized the NHID, a population database documenting health care utilization, health screening, socio-demographic variables, and mortality for the entire population of South Korea, developed and maintained by the National Health Insurance Service^11^. Our study included adults aged ≥ 19 years who were newly diagnosed with OSA between 2011 and 2018. OSA was defined as at least one claim under the International Classification of Diseases, 10th revision (ICD-10) codes for OSA (G47.33). For the control group, we selected approximately tenfold the number of age- and sex-matched adults from the NHID who had not been diagnosed with OSA. Individuals with a history of any cancer prior to the index date (the date of diagnosis of OSA or the date of the first claim between 2011 and 2018 in the NHID for the control group) were excluded. Individuals who were deceased or were diagnosed with lung cancer within 1 year after the index date were also excluded to minimize the potential effects of reverse causation. Subsequently, we conducted up to 1:5 propensity score matching by age and sex between the OSA and the control groups.

This study was conducted in accordance with the principles set out in the Declaration of Helsinki for medical research involving human subjects. Furthermore, the study was approved by the Institutional Review Board of Seoul National University Hospital (E-2111-022-1269). Informed consent was waived due to the retrospective nature of the study by the Institutional Review Board of Seoul National University Hospital.

### Study outcome and variables

The study outcome was the incidence of newly diagnosed lung cancer during the follow-up period. We determined the development of lung cancer by ICD-10 codes (C33 or C34) and registration for a special health insurance co-payment calculation in the event of cancer, introduced by the Korean government to enhance the financial protection of cancer patients by expanding National Health Insurance benefit coverage to alleviate out-of-pocket expenses for cancer patients for 5 years from the date of registration. As the differential co-payments cover 95% of medical expenses^12^, the failure to identify cancer patients from the NHID is very rare. Patients were followed up from the index date to the date of diagnosis of lung cancer, death, or the end of the follow-up (December 31, 2020), whichever came first.

We collected the following data from the NHID: age, sex, quintile of income, comorbidities including hypertension (I10 to I13, I15), type 2 diabetes (E11 to E14), ischemic heart disease (I20 to I25), heart failure (I50), atrial fibrillation or flutter (I48), transient cerebral ischemic attack or cerebral infarction (G45, I63), chronic obstructive lung disease (COPD; J44), and depression (F32, F33). These comorbidities are defined by their respective ICD-10 codes.

### Statistical analysis

Categorical variables are presented as counts and percentages, and continuous variables are presented as means with standard deviation (SD). Baseline characteristics between the OSA and control groups were compared using the independent samples *t*-test for continuous variables and the χ^2^ test for categorical variables. The incidence of lung cancer was calculated by dividing the number of events by the time at risk in person-years. To evaluate the association between OSA and risk of lung cancer, we used a Cox proportional hazards model, adjusting for potential confounding factors such as income and the presence of comorbidities. The risks for lung cancer are presented as hazard ratios (HRs) and 95% confidence intervals (CIs). We conducted subgroup analyses for the primary outcome, stratified by age and sex. Statistical significance was set at *p* < 0.05. We used SAS version 9.4 (SAS Institute, Cary, NC, USA) software for propensity score matching. Other statistical analyses were performed with R version 4.0.3 (www.r-project.org).

### Ethical approval

This study was conducted in accordance with the principles set out in the Declaration of Helsinki for medical research involving human subjects. Furthermore, the study was approved by the Institutional Review Board of Seoul National University Hospital (E-2111-022-1269). Informed consent was waived due to the retrospective nature of the study by the Institutional Review Board of Seoul National University Hospital.

## Results

### Baseline characteristics

**Figure 1:**
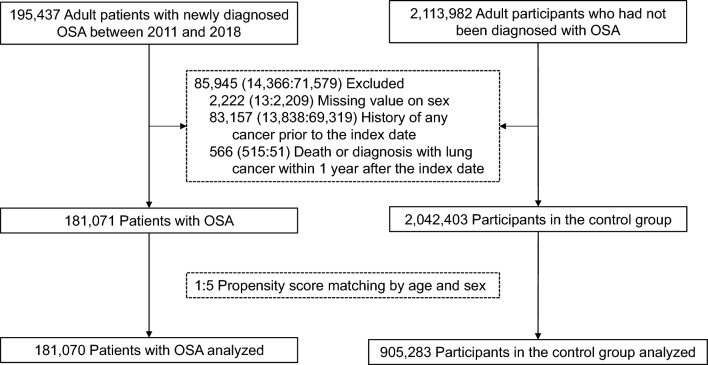
Flow diagram of the study population. OSA, obstructive sleep apnea.

A total of 195,437 adult patients newly diagnosed with OSA between 2011 and 2018 were identified in the NHID. Among these, 14,366 were excluded using the following exclusion criteria: incomplete data, a history of any cancer before the index date, or a lung cancer diagnosis or death within 1 year after the index date. Among 2,113,982 adult patients who had not been diagnosed with OSA in the same period, 71,579 were excluded using the same exclusion criteria. After up to 1:5 propensity score matching based on age and sex, 181,070 patients in the OSA group and 905,283 in the control group were analyzed (Fig. 1). The mean age of the patients was 44.6 years, and 78.1% were male. Patients with OSA had a higher prevalence of comorbidities. However, the proportion of patients in the lowest quintile of income was higher in the control group (Table 1).

**Table 1:** Baseline characteristics (*N* = 1,086,353).

Characteristics	OSA (*n* = 181,070)	Control (*n* = 905,283)	*p* value
Age, years (mean ± SD)	44.6 ± 13.6	44.6 ± 13.6	0.918
Male sex	141,429 (78.1)	707,383 (78.1)	0.765
Lowest quintile of income	27,908 (15.4)	175,900 (19.4)	< 0.001
Comorbidities			
Hypertension	72,215 (39.9)	292,410 (32.3)	< 0.001
Type 2 diabetes	55,490 (30.6)	227,138 (25.1)	< 0.001
Ischemic heart disease	29,543 (16.3)	100,685 (11.1)	< 0.001
Heart failure	9486 (5.2)	31,428 (3.5)	< 0.001
Atrial fibrillation or flutter	5424 (3.0)	153,78 (1.7)	< 0.001
Stroke	12,782 (7.1)	46,946 (5.2)	< 0.001
Chronic obstructive pulmonary disease	7877 (4.4)	33,092 (3.7)	< 0.001
Depression	27,328 (15.1)	77,933 (8.6)	< 0.001

*OSA* obstructive sleep apnea, *SD* standard deviation.

Data are presented as *n* (%) unless otherwise indicated.

### Lung cancer incidence

During follow-up over (mean ± SD) 9.1 ± 2.0 years, 2614 incident cases of lung cancer were identified among 1,086,353 patients. The mean time to lung cancer diagnosis was 6.3 ± 2.5 years from the index date. The incidence rate was 39.51 per 100,000 person-years in the OSA group, and 24.93 per 100,000 person-years in the control group (Table 1).

**Figure 2:**
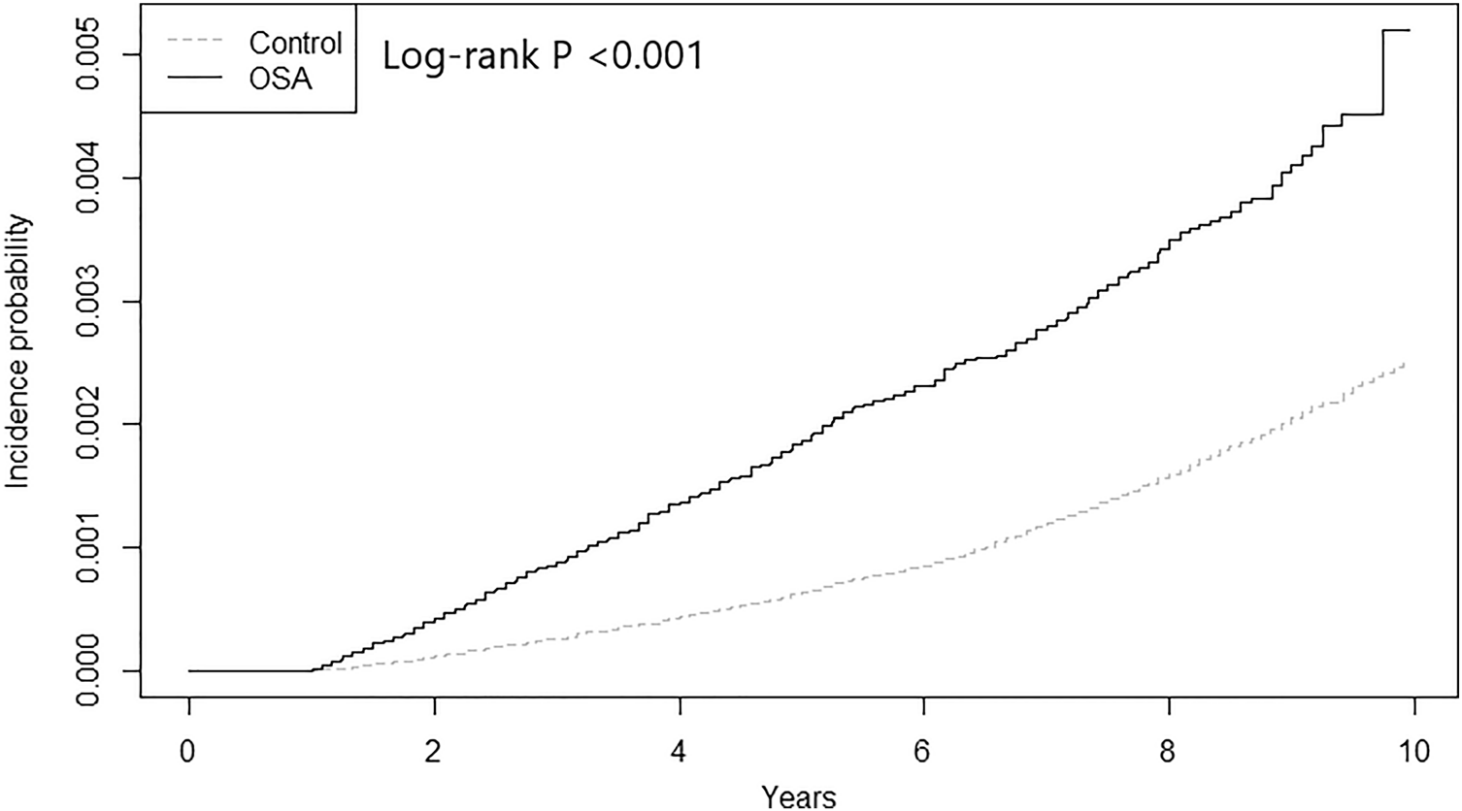
Kaplan–Meier curves of the incidence of lung cancer according to the presence of OSA. OSA, obstructive sleep apnea.

Figure 2 shows the unadjusted Kaplan–Meier curves of the incidence of lung cancer according to the presence of OSA. In an unadjusted model, patients with OSA had an increased risk of incident lung cancer (HR 2.33, 95% CI 2.09–2.60; Table 2). After adjusting for income and the presence of comorbidities including hypertension, type 2 diabetes, ischemic heart disease, heart failure, atrial fibrillation or flutter, stroke, chronic obstructive lung disease, and depression, this association remained significant (HR 1.95, 95% CI 1.74–2.18, *p*-value < 0.001; Table 2).

**Table 2:** Incidence of lung cancer according to OSA.

	*n*	Incident lung cancer	Follow-up duration, person-years	Incidence rate, per 100,000 person-years	Hazard ratio (95% CI)	Adjusted hazard ratio* (95% CI)
All						
Control	905,283	2215	8,884,557	24.93	1 (Reference)	1 (Reference)
OSA	181,070	399	1,009,909	39.51	2.33 (2.09–2.60)	1.95 (1.74–2.18)
Sex						
Female						
Control	197,900	482	1,943,380	24.80	1 (Reference)	1 (Reference)
OSA	39,641	91	219,411	41.47	2.43 (1.92–3.06)	2.14(1.69–2.70)
Male						
Control	707,383	1733	6,941,177	24.97	1 (Reference)	1 (Reference)
OSA	141,429	308	790,498	38.96	2.30 (2.03–2.61)	1.90 (1.67–2.16)
Age, years						
19–39						
Control	350,750	78	3,425,285	2.28	1 (Reference)	1 (Reference)
OSA	70,150	12	396,114	3.03	2.06 (1.10–3.85)	1.73 (0.92–3.25)
40–64						
Control	486,585	1487	4,796,128	31.00	1 (Reference)	1 (Reference)
OSA	97,317	247	544,060	45.35	2.14 (1.86–2.46)	1.86 (1.62–2.14)
≥ 65						
Control	67,948	650	663,143	98.02	1 (Reference)	1 (Reference)
OSA	13,603	140	69,134	202.51	3.25 (2.68–3.94)	2.99 (2.46–3.64)

*CI* confidence interval, *OSA* obstructive sleep apnea.

*Adjusted for income (lowest quintile or not) and the presence of comorbidities including hypertension, type 2 diabetes, ischemic heart disease, heart failure, atrial fibrillation or flutter, stroke, chronic obstructive lung disease, and depression.

The incidence rate in the female patients with OSA was 41.47 per 100,000 person-years, and that in the male patients with OSA was 38.96 per 100,000 person-years (Table 2). Kaplan–Meier curves of the incidence of lung cancer in male and female patients are shown in Supplementary Fig. 1. The adjusted HR for incident lung cancer was 2.14 (95% CI 1.69–2.70) in female patients with OSA, and 1.90 (95% CI 1.67–2.16) in male patients with OSA (Table 2). The risk of incident lung cancer increased with age (Supplementary Fig. 2). The adjusted HR for incident lung cancer was 1.73 (95% CI 0.92–3.25) in patients with OSA aged 19–39 years, 1.86 (95% CI 1.62–2.14) in those aged 40–64 years, and 2.99 (95% CI 2.46–3.64) in those aged ≥ 65 years (Table 2).

## Discussion

In this nationwide population-based cohort study utilizing Korean NHID over a mean follow-up of 9 years, we found a significant association between OSA and an elevated risk of developing lung cancer, with OSA patients demonstrating an incidence rate of 39.51 per 100,000 person-years compared with 24.93 per 100,000 person-years in the age- and sex-matched control group. OSA remained a significant risk factor for lung cancer after adjusting for various potential confounders such as income and comorbid conditions.

There is growing evidence for the putative association between OSA and cancer; however, epidemiologic studies still show inconsistent findings. In a study of a large health insurance database in the United States, the all-cancer incidence was similar between patients with OSA (defined by ICD codes) and matched controls, whereas specific cancers such as kidney, pancreas, and melanoma showed increased risks in patients with OSA^13^. Conversely, risks for some other cancers, including colorectal and prostate, were lower. Recent data from a large Australian sleep clinic cohort showed that the association between OSA (defined by apnea–hypopnea index [AHI]) and all-cancer incidence was lost after adjusting for confounders such as age, sex, body mass index, smoking status, socioeconomic status, and blood pressure. Similarly, nocturnal hypoxemia, defined as sleep time spent with an oxygen saturation < 90% (T90), was not an independent risk factor for all-cancer incidence^14^. However, according to data from a large multicenter French cohort, T90, but not AHI, was associated with all-cancer incidence after adjusting for confounders^15^. Nevertheless, the association with OSA can be heterogeneous across different cancer sites. Considering that there are specific risk factors and pathophysiology associated with each type of cancer^16^, it is crucial to reveal the association between OSA and specific cancer sites.

There is limited evidence on the associations between OSA and the risk of site-specific cancer^16,17^. Most studies have focused on all-cancer incidence as the main outcome while conducting subgroup analyses to investigate the associations with certain types of cancer^6^; however, the statistical power of subgroup analyses may be inadequate when the number of events is low^10^. A subgroup analysis of a health insurance database study in the United States showed that the incidence of lung cancer did not increase in individuals with OSA after adjustment for comorbidities^13^. This study was also limited by a relatively short mean follow-up period of less than 4 years. Similarly, another subgroup analysis of an Australian sleep clinic cohort study did not show any association between incident lung cancer and the presence of OSA or nocturnal hypoxemia^14^. However, in a subgroup analysis of a multicenter French cohort study, nocturnal hypoxemia was found to be associated with the risk of lung cancer^15^. Furthermore, in a recent meta-analysis of 4,885,518 individuals, patients with OSA had an approximately 25% higher risk of lung cancer compared with those without OSA^3^. To reduce heterogeneity, a sensitivity analysis of three studies, each with more than 5 years of follow-up, was performed. It showed that patients with OSA had a 32% higher incidence of lung cancer^3^.

Our nationwide population-based cohort study found 2614 incident cases of lung cancer among 1,086,353 patients over a mean follow-up of 9 years. To reduce the possibility of reverse causation, we excluded incident cases diagnosed in the first year of follow-up. As a result, OSA was an independent risk factor for lung cancer incidence after adjusting for demographics and comorbidities. Our study's adjusted HR for incident lung cancer was 1.95, which was higher than that found in the previous meta-analysis^3^. Differences in effect size could be explained by variations in race and follow-up duration. The previous meta-analysis included studies conducted in Western countries with relatively shorter follow-up durations. Furthermore, we found that this association was observed across both sexes and different age groups and was more prominent in women and older people. Further studies are needed to determine whether OSA may differentially affect the incidence of lung cancer in different sex and age groups.

While the exact mechanisms linking OSA and lung cancer remain to be fully elucidated, the evidence suggests that intermittent hypoxia—a hallmark feature of OSA—may play a crucial role in tumorigenesis^10,18^. Intermittent hypoxia can lead to oxidative stress, DNA damage, and systemic inflammation, creating an environment conducive to cancer development and progression via hypoxia-inducible factor^5,19^. Additionally, OSA-related sleep fragmentation may further exacerbate carcinogenesis by increasing sympathetic activation, systemic inflammation, and immune dysregulation^3,20^.

Our study has some limitations. First, we defined OSA by ICD codes without conducting sleep studies such as polysomnography, which may have introduced misclassification bias. Second, as we determined the development of lung cancer by ICD codes, and since ICD codes do not reflect pathologic subtypes of lung cancer such as adenocarcinoma, squamous cell carcinoma, and small cell lung cancer, we cannot evaluate the associations between OSA and the incidence of each subtype of lung cancer. Third, we did not measure intermittent hypoxemia or sleep fragmentation, making it difficult to evaluate any pathophysiologic link between OSA and lung cancer. Fourth, although we adjusted for demographics and comorbidities, residual confounding such as smoking, alcohol consumption, physical activity, obesity, and treatment for OSA may exist.

In conclusion, this nationwide study showed an independent association between OSA and an increased risk of lung cancer. This association between OSA and lung cancer incidence was consistent among both sexes and various age groups, with a stronger effect seen in women and older adults. These findings should be explored in further studies for a better understanding of the relationship between OSA and lung cancer, which will guide tailored prevention and management.

### Supplementary Information

Supplementary Figures.

## Data Availability

The data that support the findings of this study are available from the corresponding author upon reasonable request.
