# Supplementary material for: Association of obstructive sleep apnea with risk of lung cancer: a nationwide cohort study in Korea
Source: Sci Rep. 2024 May 29;14:12394. doi: 10.1038/s41598-024-63238-x (PMC11137051; doi:10.1038/s41598-024-63238-x)
Supplement: Supplementary file 1 — Supplementary Figures. [file 41598_2024_63238_MOESM1_ESM.pdf]

Association of obstructive sleep apnea with risk of lung cancer: A nationwide cohort study in Korea

Soomin Jo, Jaeyoung Cho

Supplementary Figure 1. Kaplan-Meier curves of incidence of lung cancer in (A) females and (B) males.

A

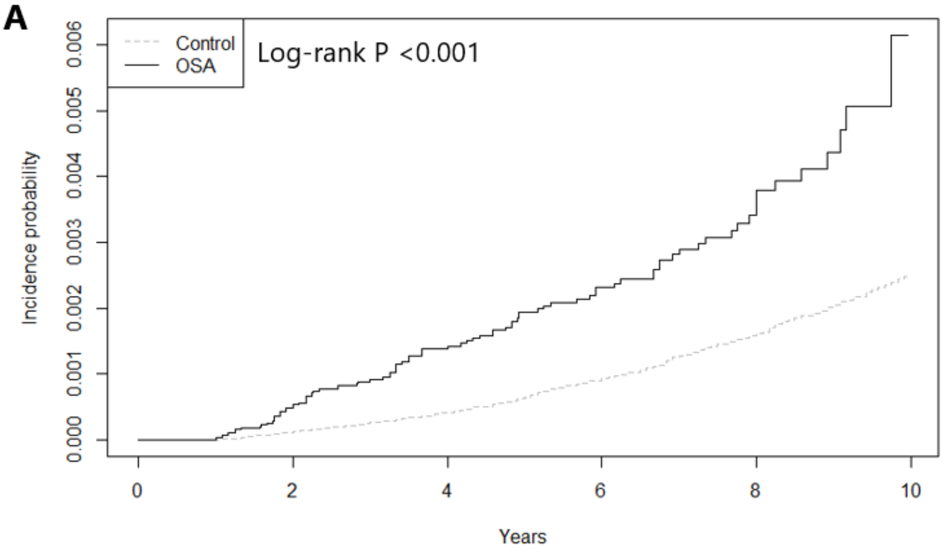

B

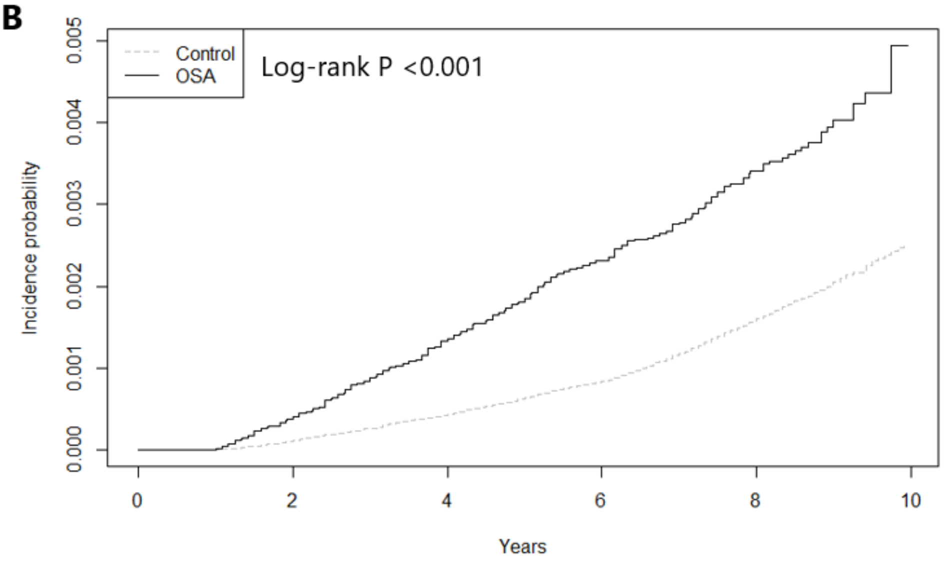

OSA, obstructive sleep apnea.

Supplementary Figure 2. Kaplan-Meier curves of incidence of lung cancer in adults aged (A) 19–39 years, (B) 40–64 years, and (C) ≥65 years.

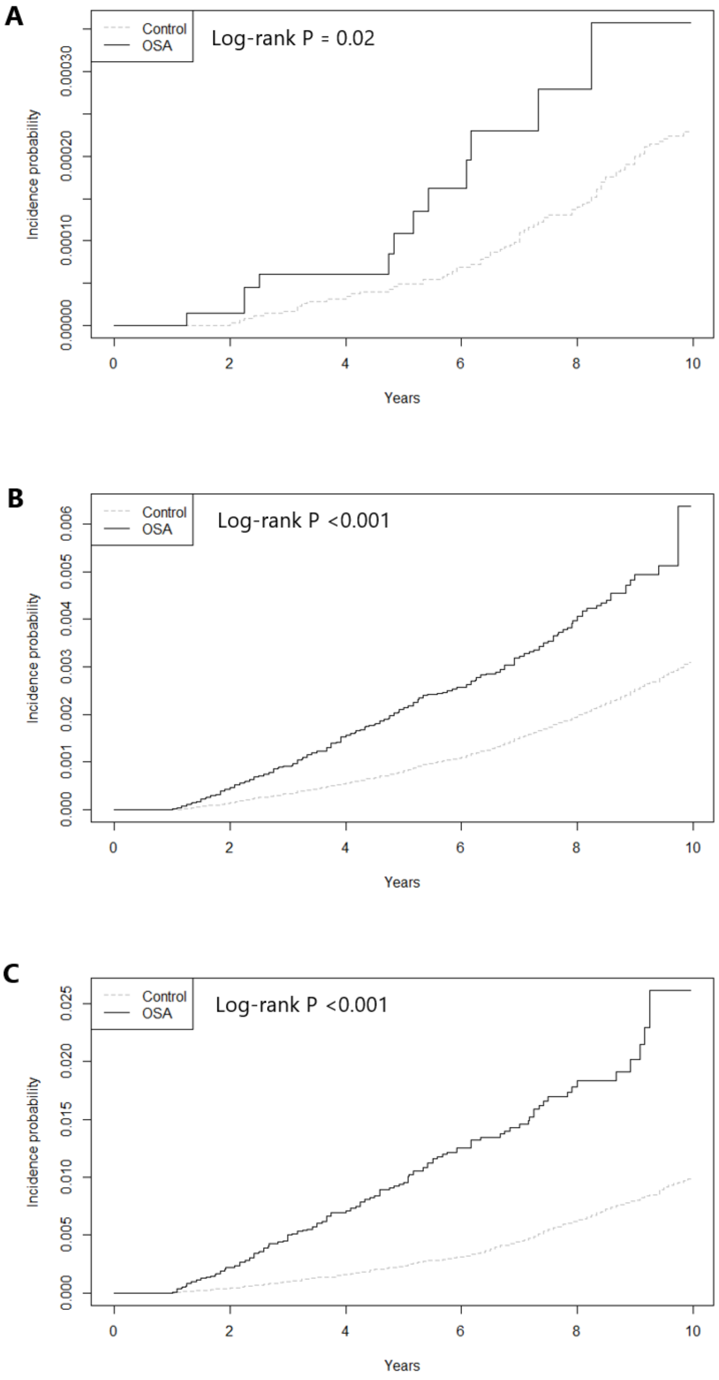

OSA, obstructive sleep apnea.
